# Opportunities and challenges of natural killer cell-derived extracellular vesicles

**DOI:** 10.3389/fbioe.2023.1122585

**Published:** 2023-03-31

**Authors:** Yuchen Qi, Xiang Zhao, Yan Dong, Min Wang, Junyi Wang, Zhichao Fan, Qin Weng, Hua Yu, Jianjun Li

**Affiliations:** ^1^ School of Medical and Life Sciences, Chengdu University of Traditional Chinese Medicine, Chengdu, China; ^2^ Department of Oncology, Southwest Hospital, Third Military Medical University (Army Medical University), Chongqing, China; ^3^ Department of General Surgery, Hospital of Chengdu University of Traditional Chinese Medicine, Chengdu, China

**Keywords:** natural killer cell, extracellular vesicles, engineering strategy, cancer immunotherapy, tumor microenvironment

## Abstract

Extracellular vesicles (EVs) are increasingly recognized as important intermediaries of intercellular communication. They have significant roles in many physiological and pathological processes and show great promise as novel biomarkers of disease, therapeutic agents, and drug delivery tools. Existing studies have shown that natural killer cell-derived EVs (NEVs) can directly kill tumor cells and participate in the crosstalk of immune cells in the tumor microenvironment. NEVs own identical cytotoxic proteins, cytotoxic receptors, and cytokines as NK cells, which is the biological basis for their application in antitumor therapy. The nanoscale size and natural targeting property of NEVs enable precisely killing tumor cells. Moreover, endowing NEVs with a variety of fascinating capabilities *via* common engineering strategies has become a crucial direction for future research. Thus, here we provide a brief overview of the characteristics and physiological functions of the various types of NEVs, focusing on their production, isolation, functional characterization, and engineering strategies for their promising application as a cell-free modality for tumor immunotherapy.

## 1 Introduction

Cancer immunotherapy has gained widespread attention as a clinically proven therapeutic strategy, and the relay transfer of natural killer (NK) cells has emerged as a promising approach to controlling the immune system against cancer. As the first line of defense against tumors and viral infections, NK cells can induce antigen-independent immune responses against malignant cells. A growing number of scientific reports and clinical studies have demonstrated that NK cell-based immunotherapy has promising antitumor effects (Laskowski et al., 2022). Furthermore, NK cell therapy or chimeric antigen receptor (CAR) NK cell therapy has unique advantages over existing hot T-cell immunotherapy (Biederstadt and Rezvani, 2021; Rafei et al., 2021). NK cells recognize and kill tumors by combining signals generated by independent inhibitory and activating receptors, effectively inhibiting tumor escape through antigen downregulation (Basar et al., 2020). Currently, a wide spectrum of research is being conducted on NK cell-related cancer therapies, including CAR NK, engineered NK cells, and allogeneic natural killer cell infusion (Tang et al., 2018; Liu et al., 2021). However, despite several clinical trials, the prospects for NK cell-based therapies for solid tumors are not optimistic. Challenges include difficulty in *ex vivo* expansion meeting clinical grade, tumor immune escape, limited *in vivo* persistence, and limited infiltration into solid tumors (Oh et al., 2019; Bald et al., 2020). Moreover, the tumor microenvironment (TME) inhibits NK cell responses (Fang et al., 2018; Lian et al., 2021). These factors directly hinder or limit the use of NK cells in solid tumor therapy.

**FIGURE 1:**
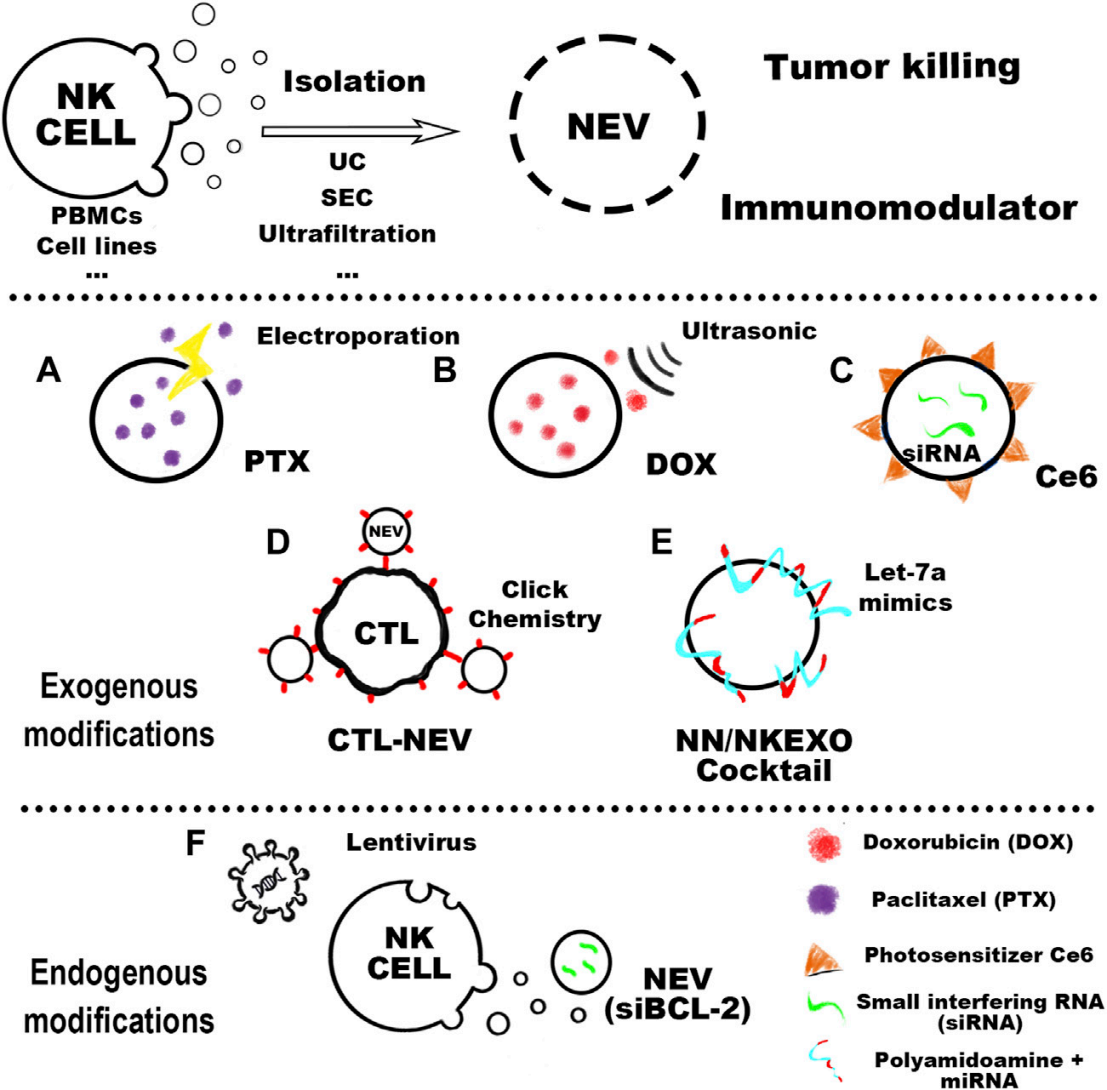
The cell sources of NEVs could be mainly divided into peripheral blood mononuclear cells (PBMC) and other cell lines. In the purification process of NEVs, the more mature isolation methods include ultracentrifugation (UC), ultrafiltration, and size exclusion chromatography (SEC). Purified NEVs have anti-tumor activity and immunomodulatory effects, and engineered modifications can confer new functions on NEVs. Common NEVs engineering techniques can be classified as exogenous and endogenous modifications. Exogenous modifications: **(A)** NEVs can be used to prepare the PTX-NEVs drug delivery system through electroporation (Han et al., 2020). **(B)** The therapeutic potential of doxorubicin-loaded NEVs shows promising antitumor activity *in vivo* against the MCF-7 induced tumor model (Pitchaimani et al., 2018). **(C)** Light-activatable silencing NK-derived exosomes (LASNEO) are orchestrated by engineering the NEVs with hydrophilic small interfering RNA (siRNA) and hydrophobic photosensitizer Ce6 (Zhang et al., 2022). **(D)** NEVs are used as a versatile toolkit to synergistically improve adoptive T-cell therapy for solid tumors (Nie et al., 2021). **(E)** NEVs are in combination with their biomimetic core–shell nanoparticles for tumor-targeted therapy (Wang et al., 2019). Endogenous modifications: **(F)** The NK cell is lentivirally transduced to express and load BCL-2 siRNAs (siBCL-2) into NEVs (20).

Extracellular vesicles (EVs) can be divided into three subgroups based on their biological origin, including exosomes (30–150 nm in diameter), microvesicles (150–1,000 nm), and apoptotic vesicles (50–2,000 nm). The role of EVs secreted by immune cells in antitumor therapy has received more attention in recent years, with many studies confirming their great potential (Shen and Ren, 2018; Choi et al., 2022a). Among them, NK cell-derived EVs (NEVs) have gained more attention for their unique biological properties. NEVs possess NK cell surface receptors and cytotoxic proteins that function similarly to parental cells, enabling them to kill tumors directly in the TME. Unlike cells, nanoscale NEVs can easily diffuse and infiltrate solid tumors and own natural targeting and biocompatibility properties. Furthermore, there have been few types of research showing that immunosuppressive TME affects NEVs. Therefore, the emergence of NEVs may overcome the limitations of NK cells in immunotherapy. Numerous studies have thoroughly investigated the biological basis for tumor killing by NEVs and found that they carry a variety of bioactive molecules, including membrane toxicity receptors, cytotoxic proteins, cytokines, and microRNAs (miRNAs). This is the biological basis for NEVs natural tumor-killing and tumor-targeting properties, as well as the ability to interact with immune cells such as tumor-associated macrophages (TAM) and cytotoxic T lymphocyte (CTL) (Federici et al., 2020). Due to the above advantages, engineered NEVs have received a lot of attention to enhance their tumor-killing capabilities. Current research focuses on the use of engineered modifications to enhance the functionality of NEVs (Yang et al., 2021a). Nevertheless, many challenges remain in the development of NEVs, such as cell source production methods and interaction mechanisms. This review aims to summarize the latest research on the production, application, mechanism, and modification of NEVs (Figure 1).

## 2 Preparation of NEVs

### 2.1 NK cell source

For both NK cells and NEVs used in therapeutic studies, the optimal source of NK cells is currently a controversial topic. Peripheral blood mononuclear cells (PBMC) and cell lines are the two main sources of NK cells used in therapeutic studies. PBMC can be obtained from patient blood or blood collected from healthy adult volunteers. Among them, PBMC-induced NK cells from cancer patients had fewer clinical adverse effects and a higher safety profile (Sakamoto et al., 2015; Fang et al., 2018; Fang et al., 2022). However, their cytotoxic properties are compromised, with significantly reduced expression of activating receptors such as NKG2D, DNAM-1, and NKp46, which directly affects their derived EVs (Cianga et al., 2021). In contrast, healthy population-derived allogeneic NK cells have high yield and cytotoxicity but a low safety profile (Federici et al., 2020). It is worth noting that PBMC-derived NK cells are difficult to use in tumor therapy on a large scale and for a long time due to the restriction of donor and blood groups (Shah et al., 2015). Another important source is NK cell lines, such as NK-92 or NK-92MI, are important sources of NK cells and have become one of the important alternatives to autologous NK cell biologics. Moreover, the NK-92 cell line is the only human NK cell line approved for clinical use by the FDA. NK-92 can be expanded in culture in the presence of cytokines (NK-92MI amplification *in vitro* is not cytokine-dependent). It is inexpensive to administer, and there is substantial evidence that it is relatively safe (Gong et al., 1994). A study comparing the distribution of cytolytic proteins in NEVs from primary NK and NK-92 cells and found strong similarities and the same satisfactory tumor-killing effect (Aarsund et al., 2022).

Furthermore, umbilical cord blood (UCB), hematopoietic stem and progenitor cells (HSPCs), induced pluripotent stem cells (iPSCs), and CAR NK cells are valuable sources of NK cells (Li et al., 2018; Kundu et al., 2021; Boyd-Gibbins et al., 2022). CAR-NK and iPSC-NK cells could benefit from advances in manufacturing and genome engineering techniques to create NK cells and NEVs with context-dependent functions and enhanced potency and specificity. Future research is required to confirm the differences in the composition and effects of EVs produced by NK cells from different sources. The common cellular sources of NEVs production and their advantages and disadvantages are summarized in Table 1.

**TABLE 1:** Advantages and disadvantages of the mainstream NK cell sources.

	PBMC (autologous)	PBMC (allogeneic)	Cell line	CAR NK**/**iPSC-NK
Advantages	Easy access and high security	High tumor-killing activity (Broader activation receptors), success in multiple clinical trials	Simple access to a large number of cells, immortality, low cost, easy to engineer (transgenic, material modifications)	Without the requirement of an autologous collection, more versatile
Disadvantages	Difficult to obtain sufficient numbers of cells, low tumor-killing activity	Difficult to obtain, expensive, and limited by donor and blood type, *in vitro* amplification weakens activity, risk of immune rejection, and graft-versus-host disease (GVHD) (Shah et al., 2015)	Safety concerns (few clinical trials), irradiation before use, general cytotoxicity, and lack of agonist receptors (Goldenson and Kaufman, 2021)	Higher risk of graft-versus-host disease (GVHD), cytokine release syndrome (CRS), low cytotoxicity after irradiation (cell line origin), and low persistence *in vivo*

### 2.2 Production of NEVs

The most common method for isolating EVs is cell culture supernatant collection. Since there is no “gold standard protocol” for the preparation of pure EVs, the properties and functions of EVs may vary depending on the culture and isolation methods. Differences in cell culture oxygen content and inoculation surface have been shown to affect EVs production (Zhang et al., 2017; Liu et al., 2020). Differences in cultural media also have an impact on EVs extraction. It has been shown that exogenous proteins introduced into cell culture can affect the type and characteristics of exosomes (Whitford and Guterstam, 2019; Chen et al., 2020). However, it has also been shown that the emergence of serum-free media modifies the biology of EVs (Mendt et al., 2018).

Obtaining EVs through natural secretion is hampered by the low yield. The advent of exosome-mimetic vesicles (EMs) with higher yield is expected to resolve this issue (Ou et al., 2021). Large-scale production of EMs could be an alternative to conventional EVs production. When cytoplasmic membranes are forced to rupture, they reassemble into smaller vesicles. Thus, EMs with diverse sizes can be produced using diverse filter membranes and micro-extruders. A study reported that the production of EMs using this method was 250 times higher than naturally secreted EVs (Lee et al., 2020). More recently, similar methods have been used to produce NK EMs, with tumor-killing abilities and impressive stability under physiological conditions, which could also be loaded with chemotherapeutic drugs for targeted cancer therapy (Pitchaimani et al., 2018; Zhu et al., 2018). However, more research is needed to investigate the differences in efficacy and safety with naturally secreted EVs.

Either artificially generated EMs or naturally secreted EVs typically contain multiple types of biological impurities. Therefore, it is essential to ensure that the purified products are inherently EVs without other contaminants before performing any functional analysis of the EVs. The current EVs isolation and purification methods and their advantages and disadvantages are summarized in Table 2.

**TABLE 2:** The advantages and disadvantages of the mainstream EVs isolation method.

Isolation method	Advantages	Disadvantages
Differential ultracentrifugation (UC)	Easy to use, high productivity, and low requirement for technical expertise without complex sample pre-treatment Coughlan et al. (2020)	The process is time-consuming, recovery rates vary widely, reproducibility is poor, and purity is not high. Langevin et al. (2019), Yang et al. (2020)
Density gradient ultracentrifugation	Providing a purer sample for subsequent applications Konoshenko et al. (2018)	The process requires not only expensive equipment but also trained technicians. In addition, since density gradient ultracentrifugation depends only on the density difference between different solutes in the sample, the method cannot separate substances with densities similar to those of EVs, and its capacity is largely limited by the narrow loading zone Li et al. (2017)
Ultrafiltration	It is an ideal alternative to classic ultracentrifugation strategies, due to the short separation times, high throughput, and the ability to customize the selection of sample subpopulations by adjusting the pore size of the screen. Heinemann and Vykoukal (2017), Yu et al. (2018)	The clogging of the filter by vesicles could lead to high experimental costs and low separation yields. In addition, the ultrafiltration process may deform the vesicles and fail to remove contaminants of similar size to the target product Chen et al. (2020)
Size exclusion chromatography (SEC)	The purity of the isolated sample is high and its natural biological activity can be maintained to a large extent. Moreover, the sample requirement is low, and the screening pore size can be adjusted Ma et al. (2019)	EVs prepared by SEC columns are usually time-consuming and costly, it exhibits a wider size distribution, especially in the smaller size range, indicating the presence of contaminants of similar size to EVs. Guo et al. (2021)

The advantages and disadvantages of each option described above. The results of each isolation method used to isolate EVs from different cell sources may vary (Yang et al., 2020). Differential ultracentrifugation is currently the primary method of NEVs isolation, but a growing number of studies have shown that employing multiple centrifugation techniques simultaneously yields better results (Patel et al., 2019).

### 2.3 Storage of NEVs

As a promising cell-free therapy, achieving long-term and stable storage of NEVs plays a key role in their clinical application. Therefore, it is necessary to explore preservation techniques to protect the biological activity of NEVs for transport and clinical applications. Common conservation techniques include freezing, lyophilization, and spray drying (Kusuma et al., 2018; Charoenviriyakul et al., 2019; Zhang et al., 2020a). Any frozen storage may “frostbite” the EVs, and the use of antifreeze may extend their shelf life. The traditional approach of adding DMSO during cryopreservation can protect the biological activity of EVs (Wu et al., 2015). Furthermore, alginate is considered as the most effective disaccharide antifreeze agent and prevents EVs aggregation and increases its stability without changing EVs morphology (Bosch et al., 2016; Charoenviriyakul et al., 2018). A recent study reported that PBS supplemented with human albumin and trehalose buffer significantly improved the short and long-term preservation of EVs samples stored at −80°C, and maintained stability over multiple freeze-thaw cycles (Gorgens et al., 2022). Moreover, the storage of EVs also varies from different sources and modifications (Agrawal et al., 2017). Multiple studies on NEVs have analyzed the impact of storage on NEVs and concluded that the existing technology could effectively ensure the storage stability of NEVs (Jong et al., 2017; Farcas and Inngjerdingen, 2020). In summary, the rational use of various EVs storage methods can significantly improve the storage stability of EVs and provide greater application benefits.

## 3 Function mechanisms of NEVs

In recent years, as research on NEVs have continued, knowledge about the mechanisms underlying their function has been gained. Several studies have confirmed the ability of NEVs to target and kill tumor cells, which have been summarized in Table 3. This section highlights the characteristics and mechanisms of the currently known NEVs in oncology therapy.

**TABLE 3:** Existing studies for NEVs.

Cell source	Size(nm)	Isolation method	Engineering strategy	Cytolytic activity (cells)	Year of publication	References
Human PBMCs	40–100	UC		K562; Jurkat; PHA-activated PBMCs	2012	Lugini et al. (2012)
Human PBMCs	40–150	UC		SK-N-SH; CHLA-255	2017	Shoae-Hassani et al. (2017)
Human PBMCs	50–200	PEG8000 precipitation and dialysis		NALM-6, SupB15, CHLA255	2017	Jong et al. (2017)
NK-92MI cells	100–150	UC		B16F10	2017	Zhu et al. (2017)
NK-92MI cells	118 ± 33.1	UC		D54/F	2018	Zhu et al. (2018)
NK-92 cells	190–460	UC			2018	Korenevskii et al. (2018)
NK-92 cells	88 ± 1	density gradient ultracentrifugation	Load with Dox	MCF-7	2018	Pitchaimani et al. (2018)
Human PBMCs	Mean 92.45	SEC		MYCN-amplified CHLA-136 and LAN-5	2019	Neviani et al. (2019)
NK-92MI cells	106.9 ± 21.6	UC		U87-MG	2019	Zhu et al. (2019)
Human PBMCs	Mean 100	UC	Load with nanomaterials and therapeutic miRNAs	MDA-MB-231. CHLA-255	2019	Wang et al. (2019)
Human PBMCs	60–150	UC		Mia PaCa-2; PANC-1	2019	Sun et al. (2019)
Human PBMCs	Exo: 124 ± 3.8	UC			2020	Federici et al. (2020)
	MV: 315.2 ± 4.8					
Human PBMCs	135.9 ± 0.5	UC		NALM-18	2020	Di Pace et al. (2020)
Human PBMCs		UC		HepG2; SW-620; MKN-74; MCF-7; T98G	2020	Choi et al. (2020)
NK-92 cells	Mean 100	UC	Load with paclitaxel	MCF-7	2020	Han et al. (2020)
NK3.3	133–193	Exo Quick-TC (SBI), UC		K562, Jurkat, MDA-MB-231, MCF7	2021	Cochran and Kornbluth (2021)
NK-92MI cells	80–130	The anti‐CD63 conjugated magnetic beads		Patient-derived circulating tumor cell lines in non-small cell lung cancer	2021	Kang et al. (2021)
NK-92 cells	Mean 100	UC	Combined with CTL	B16-OVA	2021	Nie et al. (2021)
NK-92MI cells	30–150	centrifugal filters and Exosome Purification kit	Endogenous loading BCL-2 siRNAs (siBCL-2)	ER^+^ MCF-7, T-47D, MCF-10A	2021	Kaban et al. (2021)
NK-92MI cells	Mean 120	UC	Load with hydrophilic siRNA and the hydrophobic photosensitizer Ce6	HepG2-Luc, CT26, RAW264.7	2022	Zhang et al. (2022)
Human PBMCs	165–209	UC			2022	Dosil et al. (2022)
NK-92MI cells	100–130	UC		Hep3B, HepG2, Huh7	2022	Kim et al. (2022)

### 3.1 NEVs exert antitumor effects through their contents and membrane proteins

**FIGURE 2:**
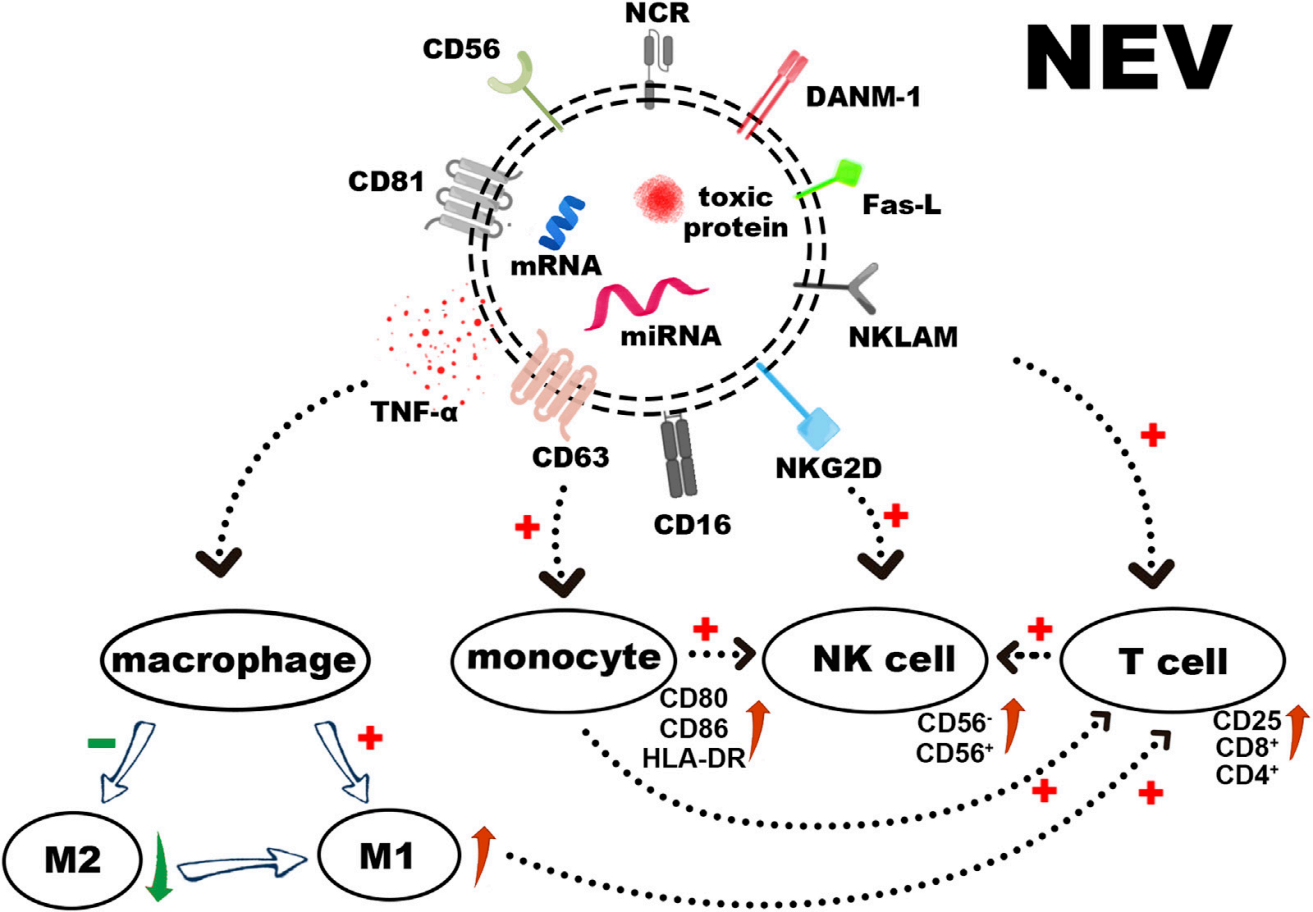
The surface receptors of NEVs, loaded cytotoxic proteins, and functional miRNAs induce apoptosis in tumor cells. In addition to stimulating the polarization of macrophages towards M1 and activating T cells directly or *via* activated monocytes, NEVs can also activate resting NK cells, thereby augmenting their tumor-killing ability.

NEVs contain many substances acting as tumor killers, such as membrane proteins, toxic proteins, and miRNAs. In this section, we will elaborate on each section individually (as shown in Figure 2).

Several studies have shown that NEVs can express NK cell surface receptors (Zhu et al., 2019; Choi et al., 2020). These receptors include Natural Killer Lytic-Associated Molecule (NKLAM), Fas-L, DNAX accessory molecules-1 (DNAM-1/CD226), and NKG2D (CD314) (Enomoto et al., 2021). The expression of the natural cytotoxic receptor (NCR), NKp44 (CD336), NKp30 (CD337), NKp46 (CD335), and CD16 varies according to cell sources and activation status (Lugini et al., 2012). Moreover, NEVs induce apoptosis through a classical ligand/receptor interaction between Fas-L on the membrane surface and Fas on the target cell membrane. Fas-L binding to the membrane receptor results in the formation of the death-inducing signaling complex (DISC), which activates the extrinsic apoptotic pathway by activating the caspase pathway (Lavrik and Krammer, 2012; Sparrow and Bodman-Smith, 2020). The CD47 expressing on the NEVs surface interacts with its receptor on macrophages, SIRP-α, to inhibit the elimination of NEVs by macrophage *via* phagocytosis, thus enabling longer cycle times (Jaiswal et al., 2009; Wang et al., 2019).

Perforin, granzyme A, B, granulysin, and tumor necrosis factor α (TNF-α) are all found in NEVs (Korenevskii et al., 2018; Cochran and Kornbluth, 2021). The perforin in NEVs can penetrate the cell membrane and allow cytotoxic proteins (granzyme A, B, granulysin) to enter the target cell and induce apoptosis by disrupting the outer mitochondrial membrane potential and cleaving caspases (Leon et al., 2017; Wu et al., 2019). Among them, granzyme B targets and cleaves cystathionine-3 and -7 directly, leading to the rapid initiation of apoptosis. It also induces an intrinsic apoptotic pathway by cleaving Bid to tBid (BH3 interacting domain death agonist protein), which disrupts the outer mitochondrial membrane potential and releases cytochrome C (MacDonald et al., 1999). The specific target of granzyme A in the apoptotic pathway is the SET complex, an ER-associated complex whose cleavage causes single-stranded DNA damage (Lieberman, 2010). Granulysin can induce apoptosis by binding to target cell membranes through electrostatic interactions based on its positive N-terminal charge. This process can disrupt cell membranes, active Caspase-9, and Caspase-12 by damaging mitochondria, as well as damaging the endoplasmic reticulum and activating Caspase-7 (Sparrow and Bodman-Smith, 2020). One quantitative analysis study demonstrated that NK-92 EVs revealed higher levels of perforin and Fas-L than NK cells and performed more effective inhibition of tumor proliferation (Zhu et al., 2017).

Regulatory miRNAs found in NEVs demonstrate tumor-killing and immunomodulation ability. Among these, miR-3607-3p encapsulated in NEVs inhibits cancer cell migration and invasion; miR-3607-3p-enriched NEVs may inhibit the malignant transformation of pancreatic cancer by directly targeting IL-26, and decreased miR-3607-3p levels were associated with poor prognosis and tumor metastasis (Sun et al., 2019). Another study demonstrated that miR-186-5p in NEVs can inhibit the growth and spread of neuroblastoma and induce apoptosis, and miR-186-5p containing NEVs was also taken up by NK cells to reduce the inhibition of cytotoxicity by the TME (Neviani et al., 2019). A recent study suggests that miR-10b-5p, miR-92a-3p, and miR-155-5p found in NEVs play a crucial role in immune regulation (Dosil et al., 2022). In addition, more information was summarized in Table 4.

**TABLE 4:** The functions of miRNAs contained in NEVs.

miRNAs	Functions	References
miR-3607-3p	This miRNA inhibits cancer cell migration and invasion	Sun et al. (2019)
miR-186-5p	This miRNA impairers neuroblastoma tumor growth and inhibits tumor immune escape by targeting the TGF-β pathway	Neviani et al. (2019)
miR-92a, miR-155	These miRNAs promote IFN-γ production	Dosil et al. (2022)
miR-10b-5p, miR-92a-3p	These miRNAs promote GATA3 downregulation and subsequent T-bet de-repression, reprogramming recipient T cells towards the Th1 phenotype	Yu and Kim (2020), Dosil et al. (2022)
miR-207	This miRNA alleviates depression-like symptoms in mice	Li et al. (2020)
miR-122-5p, miR-409-3p, and miR-451a	These miRNAs demonstrate protein translational modifications dependent mechanism of miRNA-specific shuttling into NEVs	Dosil et al. (2022)
miR-20a-5p, miR-25-3p	These miRNAs are transferred through the immune synapse, with an impact on germinal center reaction and antibody production	Fernandez-Messina et al. (2020)

### 3.2 Immunomodulatory effects of NEVs

**FIGURE 3:**
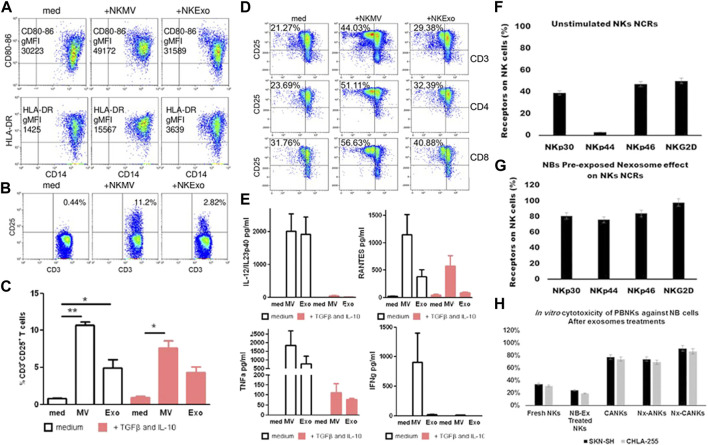
**(A)** NEVs activate monocytes. Flow cytometry analysis of CD80–CD86 geo mean fluorescence intensity (gMFI) of gated CD14^+^ cells in PBMCs cultured in the presence or absence of NEVs, and/or lipopolysacharide (LPS) for 24 h. Upper panels: Representative dot plots showing CD80–CD86 expression in the presence of NEVs, lower panel: Flow cytometry of human leukocyte antigen DR isotype (HLA-DR) gMFI of CD14^+^ gated monocytes (Federici et al., 2020). **(B)** Flow cytometry analysis of CD25 expression by CD3^+^ gated T cells in PBMCs evaluated after 72 h of culture with NEVs (Federici et al., 2020). **(C)** The graph shows the results obtained with PBMCs of different healthy donors (*n* = 3), in the presence or absence of transforming growth factor beta (TGFβ)/interleukin (IL)-10 (10 ng/ml each) (Federici et al., 2020). **(D)** NEVs affect the interaction between monocytes and T cells. Flow cytometry analysis of 72 h proliferation and CD25 expression by CD3, CD4, and CD8 T cells cultured in the presence of monocytes (medium), monocytes preconditioned with NEVs (Federici et al., 2020). **(E)** NEVs induce the release of cytokines by PBMCs. Cytometric bead array-measured cytokine production of 72 h PBMCs cultured (Federici et al., 2020). **(F–H)** Activation of resting NK cells by NEVs affects the expression of natural cytotoxicity receptors on their surface and tumor-killing viability (Shoae-Hassani et al., 2017). **(F)** NK cells were stained with specific NCRs monoclonal antibodies, a resting NK cell expresses different levels of NCRs. **(G)** The NEVs induce the expression of NCRs especially NKp44 similar to cytokine-activated NK. **(H)** *In vitro* cytotoxicity of peripheral blood natural killer cells against neuroblastoma (NB) cells. NEVs strongly stimulated NK activity in the presence of IL-21.

EVs secreted by numerous immune cells can be used to regulate innate and acquired immune responses (Lugini et al., 2012; Hong and Kim, 2022). NEVs possess similar immunomodulatory functions in the immune system (Figure 2). On the one hand, NEVs can effectively reduce the number of pre-tumor M2 macrophages or induce tumor-killing M1 macrophage polarization, which attenuates TAM-mediated CTL inhibition *via* the change of TAM -secreted cytokines and membrane surface proteins, and induces a direct antitumor effect of M1 macrophage (Bellora et al., 2014; Jia et al., 2020; Nie et al., 2021). NEVs can also act directly on T-cell activation or indirectly by stimulating monocytes to positively influence T-cell activation (Figures 3A–E) (Federici et al., 2020). Analyzing the miRNA types in NEVs reveals that they promote T-cell activation and induce DC expression of MHC-II and CD86 (Dosil et al., 2022). Furthermore, NEVs contribute to NK cell activation. A study demonstrated that NEVs pre-exposed to tumor cells could activate resting NK cells in humans, leading to higher levels of NCR and acquiring greater tumor-killing capacity (Figures 3F–H) (Shoae-Hassani et al., 2017). However, the immunomodulatory function of NEVs is still unknown due to the lack of in-depth mechanistic studies.

## 4 Characteristics of NEVs for therapeutic

### 4.1 Penetration

The TME and biological barrier, which are difficult to overcome in traditional tumor therapy, are important reasons that affect the therapeutic effect. NEV has a smaller molecular diameter and greater tissue penetration into solid tumors compared to whole cells. For example, the small molecular size of NEVs allows them to easily cross the blood-brain barrier (BBB) and enter the cancer reservoir to kill or deliver the drugs to central nervous system (CNS) tumors (Neviani et al., 2019; Weng et al., 2021; Choi et al., 2022b).

### 4.2 Natural targeting

Multiple studies have confirmed the ability of NEVs to target tumors (Lugini et al., 2012; Zhu et al., 2019; Sayitoglu et al., 2020). This characteristic may be attributed to the membrane proteins CXC receptors (CXCR3 and CXCR4), NCR, NKG2D, and DNAM-1 on the surface of NEVs, which can induce cancer cell lysis while targeting tumor cells (Deng et al., 2018; Wang et al., 2019; Di Pace et al., 2020; Sayitoglu et al., 2020; Aarsund et al., 2022). However, there is still some controversy about the targeting mechanism of NEVs.

### 4.3 Biocompatibility

NEVs enter target cells through micropinocytosis, and the number of internalized NEVs correlates with tumor cell cytotoxicity (Azarmi et al., 2020; Di Pace et al., 2020; Enomoto et al., 2021). It was discovered that co-incubating NEVs with target cells for approximately 30 min resulted in detection in target cells and induced maximal cytotoxic effects after 8–14 h (Zhu et al., 2017; Di Pace et al., 2020). Furthermore, it has been demonstrated that the acidic tumor microenvironment promotes the uptake of NEVs by tumor cells (Parolini et al., 2009; Fais, 2013).

### 4.4 Security

All current NEVs research has addressed the safety of NEVs at the cellular or animal level. Some studies point out that NEVs derived from PBMC have cytolytic activity against cancer cells but not against normal resting PBMC cells or normal cells (Lugini et al., 2012; Groot Kormelink et al., 2018). Furthermore, numerous studies have concluded that NEVs do not pose serious safety concerns in animal studies (Han et al., 2020; Kaban et al., 2021; Zhang et al., 2022). Existing research can provide initial confirmation of the safety of NEVs. However, because most of them are *in vitro* experiments, safety issues will become an issue that has to be addressed for the future development of NEVs.

### 4.5 Adjustability

EVs-secreting behavior of NK cells is independent of cell activation status (Fais, 2013; Aarsund et al., 2022). However, the killing activity of NEVs is closely related to the state of cell activation (Lugini et al., 2012). This process is regulated by many factors; for example, NEVs exhibited stronger cytotoxic effects and elevated levels of cytotoxicity-related molecules in hypoxic environments (Jiang et al., 2021). NEVs pre-exposed to the tumor environment may have higher cytotoxicity (Shoae-Hassani et al., 2017). Furthermore, the cytotoxicity of NEVs can be modulated by various cytokines (Markova et al., 2021; Aarsund et al., 2022). Namely, higher quality NEVs would be produced by appropriate regulation of NK cells based on the above characteristics in the future.

## 5 Engineering strategy for NEVs

Endowing EVs with a variety of fascinating capabilities *via* common engineering strategies has become a crucial direction for much applied research. Common EVs engineering techniques can be classified as endogenous and exogenous modifications. The primary objective is to increase the targeting ability or transform them into drug carriers (Gudbergsson et al., 2019; Zhang et al., 2020b). Existing engineering studies of NEVs are few but have shown satisfactory results.

### 5.1 Exogenous modifications

**FIGURE 4:**
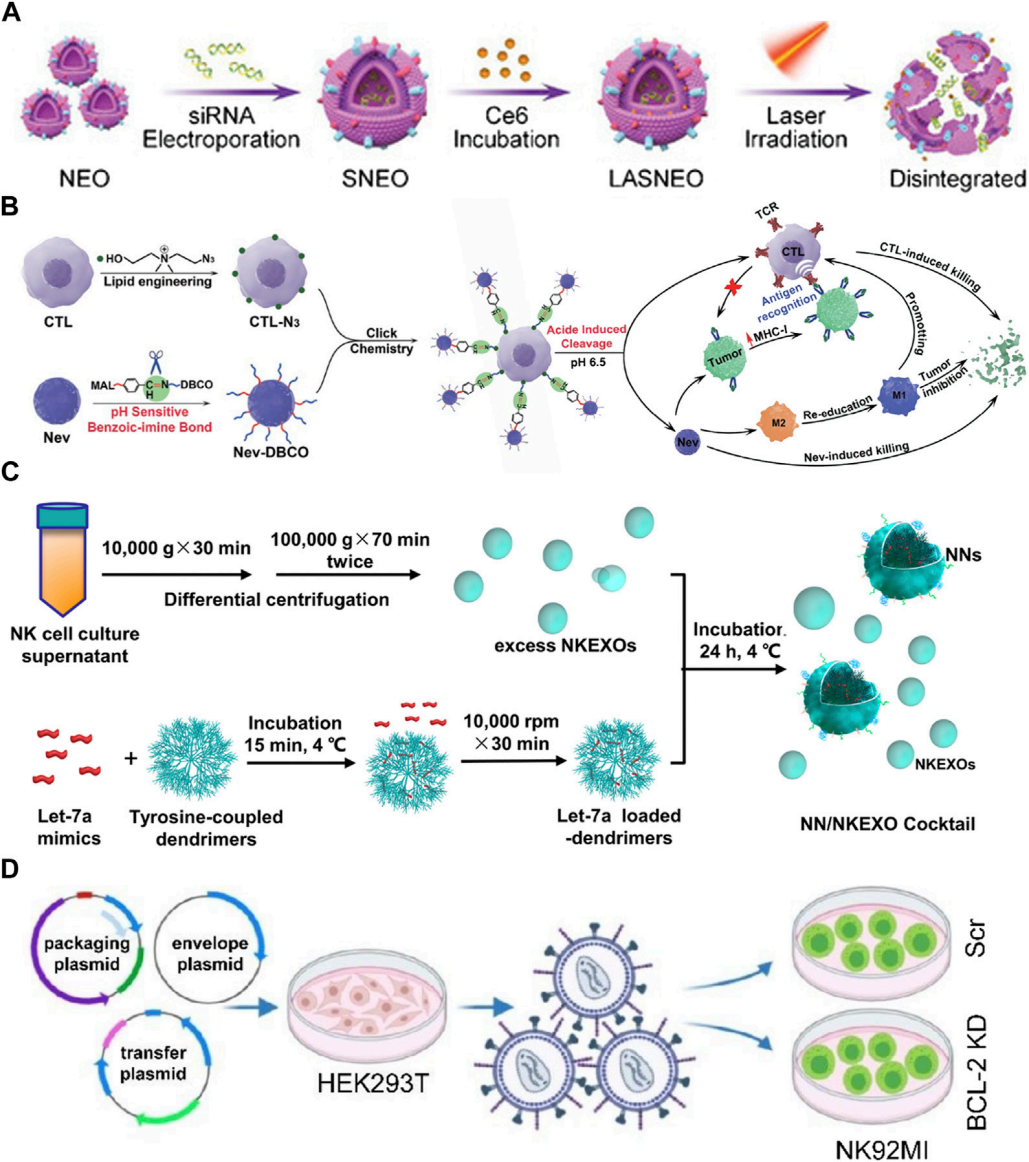
**(A)** Schematic illustration of light-activatable silencing NK-derived exosomes (LASNEO) mediated synergetic tumor eradication. **(B)** Binding of NEVs to CLTs *via* click chemistry reaction. **(C)** Schematic design of the NN/NKEXO cocktail for tumor targeting and drug delivery. **(D)** Loading of BCL-2 siRNAs (siBCL-2) in NK-92MI-derived EVs by lentiviral transfection.

NEVs are suitable for drug delivery systems (DDS) due to their strong penetration, antitumor activity, and natural targeting. The current strategy is transporting exogenous drugs into NEVs, with engineering methods mainly including electroporation, ultrasonication, extrusion, freeze-thaw cycles, and saponin treatment (Thakur et al., 2022). NEVs were used in a study to enhance the antitumor effects of the drug by encapsulating paclitaxel *via* electroporation (Figure 1A) (Han et al., 2020). Another study reported that NEVs loaded with doxorubicin by ultrasonication demonstrated excellent antitumor activity against MCF-7 human breast cancer cells both *in vitro* and *in vivo* (Figure 1B) (Pitchaimani et al., 2018). A recent study using NEVs loaded with hydrophilic siRNA and the hydrophobic photosensitizer Ce6 showed obvious tumor-killing effects due to not only the anti-tumor property of the NEVs but also the combination of the powerful gene silencing effect by the delivery of siRNA and significant photodynamic therapeutic effects with reactive oxygen species (ROS) generated after laser irradiation (Figures 1C, 4A) (Zhang et al., 2022). The use of NEVs in drug delivery overcomes most of the drawbacks of conventional nanomaterial drug delivery systems.

Surface engineering is another type of exogenous modification of interest. Surface engineering of NEVs can improve their targeting or binding to other substances, increasing their stability and duration of action *in vivo*. Introducing nanomaterials and inserting lipophilic components into the membrane by fusion with liposomes or adsorbing molecules is the main approach for the surface engineering of NEVs (Yang et al., 2021a). The linkage can also be formed through covalent bonds on the vesicle surface through azide-alkyne cycloaddition reactions (Richter et al., 2021). In a recent study, NEVs were modified with dibenzocyclooctynes (DBCO), and CTL was modified with azide groups, respectively, which were subsequently linked *via* biorthogonal chemistry. Due to the pH-responsive structure, the NEVs could be released at low PH, exploiting their ability to target tumors during circulation and promote CTL to kill tumors (Figures 1D, 4B) (Nie et al., 2021). Another study reported using cocktail therapy by combining NEVs with dendrimer core loaded with therapeutic miRNAs for tumor-targeted therapy (Figures 1E, 4C) (Wang et al., 2019).

### 5.2 Endogenous modifications

Endogenous modification is also an essential method for the functionalization of NEVs. It is intended to engineer the membrane and contents of NEVs by genetically modifying the parent cells expressing the specific target product or chimeric protein. A genetic engineering study of the NK-92MI using lentiviral transduction to express BCL-2 siRNA (siBCL-2), which is enriched in NEVs and successfully enhanced tumor-killing ability by inhibiting overexpression of BCL-2 in breast cancer (Figures 1F, 4D) (Kaban et al., 2021). A study reported that EVs isolated from mesothelin-targeted CAR-T cells maintained most of the parental cells’ characteristics and had the same therapeutic potential without significant side effects (Yang et al., 2021b). Moreover, the administration of CAR cell-derived EVs is relatively safer than CAR cell therapy (Fu et al., 2019). However, there still exists no detailed study using CAR NK-derived EVs. Take as a whole, genetic engineering enables good control over the generated EVs; once the corresponding cell line is established, no further work is required to generate the modified EVs, making it an ideal method for the mass production of engineered EVs in the future.

## 6 Discussion

This review highlights the challenges and potential of NEVs in cancer therapy, which has demonstrated tremendous advantages in recent years as an emerging cell-free therapy in cancer immunotherapy, including smaller size, greater tissue penetration, lower acquisition costs, and independence from inhibitory TAM compared to conventional NK cell therapies. The NEVs inherit the tumor-killing and natural targeting abilities of their parent cells. It is associated with relatively few immune side effects due to the absence of cellular involvement. Therefore, in addition to cell therapy, NEVs have the potential to play a crucial role in future tumor immunotherapy.

The role of NEVs is still poorly understood, and researchers continue to investigate it. One study showed that NK-92MI cells-derived EVs could inhibit TGF-β1-induced HSC proliferation and activation, preventing liver fibrosis by carrying miR-223 (Wang et al., 2020a; Wang et al., 2020b). Another study demonstrated that miR-207-containing NEVs alleviated symptoms of chronic mild stress in mice, suggesting that NEVs may also have a role in the treatment of depression (Wang et al., 2020a; Wang et al., 2020b). Moreover, NEVs ameliorated lung injury in a mouse model of *Pseudomonas aeruginosa* lung infection by promoting M1 macrophage polarization. This suggests that NEVs may play a protective role in inflammation, especially in diseases with an imbalanced M1/M2 macrophage ratio (69). As the in-depth functions of NEVs have not yet be investigated, following research may need to focus on the functional contained biomolecules and the critical roles in the immune regulation process.

Despite the large number of studies demonstrating the efficacy of NEVs in cancer therapy, the development of NEVs still faces significant challenges. Not only do NEVs face these challenges, but all therapeutic EVs developments must also overcome them. The first is the heterogeneity of EVs, which complicates quality control and hinders a comprehensive understanding of their function. The main reason comes from the cell source and isolation methods for the production of EVs. The optimal cell source and isolation method for EVs is still under investigation. Ultracentrifugation is the most widely-used EVs isolation method, which needs to be integrated with another isolation method to improve the separation purity. The second challenge is selecting designs that improve the cycling stability and the cytotoxicity of NEVs. As mentioned above, although various endogenous and exogenous modification methods are used, there is no effective method to improve the loading efficiency of bioactive molecules without compromising the integrity of EVs, and most modification methods may cause clustering. Furthermore, it is necessary to evaluate the need for these modifications and their improvement in therapeutic efficacy. A reliable method to determine whether the loaded EVs contain active molecules is still urgently needed (Wahlgren et al., 2012). Finally, due to the insufficient number of studies and inconsistent experimental conditions, there is no uniform standard on how numerous NEVs and how long it will take to achieve the desired anticancer effect, as well as what delivery method and treatment regimen should be employed during treatment to achieve improved clinical outcomes. Therefore, there is still much work to be done before the utilization of NEVs in clinical settings. Although the full-scale mechanism and function of NEVs need to be addressed, NEVs are a highly promising cell-free therapeutic option, which are easily to be obtained, modified, and stored in comparison with cells. The increasing number of studies on engineering NEVs have also proved it as an excellent vector for personalized modification. The unique anti-tumor properties of NEVs convince us that the anti-tumor strategy based on NEVs is worthy of comprehensive and in-depth study. Future research should take full advantage of NEVs and integrate it with multiple therapeutic strategies including sonodynamic therapy, photodynamic therapy, photothermal therapy and radiotherapy, so as to achieve more powerful tumor-killing effects. We hope this review will contribute to the promotion of multidisciplinary research on NEVs in a concerted effort to make NEVs the next-generation of cancer therapeutic strategy.

## Abbreviations

BBB, blood-brain barrier; CAR, chimeric antigen receptor; CNS, central nervous system; CTL, cytotoxic T lymphocyte; DBCO, dibenzocyclooctynes; DDS, drug delivery systems; EMs, exosome-mimetic vesicles; EVs, Extracellular vesicles; HSPCs, hematopoietic stem and progenitor cells; iPSCs, induced pluripotent stem cells; miRNAs, microRNAs; NCR, natural cytotoxic receptor; NEVs, natural killer cell-derived EVs; NK, natural killer; NKLAM, Natural Killer Lytic-Associated Molecule; PBMC, peripheral blood mononuclear cells; ROS, reactive oxygen species; siRNA, small interfering RNA; TAM, tumor-associated macrophages; TME, tumor microenvironment; TNF-α, tumor necrosis factor α; UCB, umbilical cord blood.

## References

[B1] Aarsund M. Segers F. M. Wu Y. Inngjerdingen M. (2022). Comparison of characteristics and tumor targeting properties of extracellular vesicles derived from primary nk cells or nk-cell lines stimulated with il-15 or il-12/15/18. Cancer Immunology Immunotherapy.71 (9), 2227-2238. 10.1007/s00262-022-03161-0 35119498 PMC9374793

[B2] Agrawal A. K. Aqil F. Jeyabalan J. Spencer W. A. Beck J. Gachuki B. W. (2017). Milk-derived exosomes for oral delivery of paclitaxel. Nanomedicine 13 (5), 1627–1636. Epub 2017/03/17. 10.1016/j.nano.2017.03.001 28300659

[B3] Azarmi M. Maleki H. Nikkam N. Malekinejad H. (2020). Transcellular brain drug delivery: A review on recent advancements. Int. J. Pharm. 586, 119582. Epub 2020/07/01. 10.1016/j.ijpharm.2020.119582 32599130

[B4] Bald T. Krummel M. F. Smyth M. J. Barry K. C. (2020). The nk cell-cancer cycle: Advances and new challenges in nk cell-based immunotherapies. Nat. Immunol. 21 (8), 835–847. Epub 2020/07/22. 10.1038/s41590-020-0728-z 32690952 PMC8406687

[B5] Basar R. Daher M. Rezvani K. (2020). Next-generation cell therapies: The emerging role of car-nk cells. Hematol. Am. Soc. Hematol. Educ. Program 2020 (1), 570–578. Epub 2020/12/05. 10.1182/hematology.2020002547 PMC7727537 33275752

[B6] Bellora F. Castriconi R. Dondero A. Carrega P. Mantovani A. Ferlazzo G. (2014). Human nk cells and nk receptors. Immunol. Lett. 161 (2), 168–173. Epub 2013/12/24. 10.1016/j.imlet.2013.12.009 24361820

[B7] Biederstadt A. Rezvani K. (2021). Engineering the next generation of car-nk immunotherapies. Int. J. Hematol. 114 (5), 554–571. Epub 2021/08/29. 10.1007/s12185-021-03209-4 34453686 PMC8397867

[B8] Bosch S. de Beaurepaire L. Allard M. Mosser M. Heichette C. Chretien D. (2016). Trehalose prevents aggregation of exosomes and cryodamage. Sci. Rep. 6, 36162. Epub 2016/11/09. 10.1038/srep36162 27824088 PMC5099918

[B9] Boyd-Gibbins N. Karagiannis P. Hwang D. W. Kim S. I. (2022). Ipscs in nk cell manufacturing and nkev development. Front. Immunol. 13, 890894. Epub 2022/07/26. 10.3389/fimmu.2022.890894 35874677 PMC9305199

[B10] Charoenviriyakul C. Takahashi Y. Nishikawa M. Takakura Y. (2019). Erratum to ‘Preservation of exosomes at room temperature using lyophilization. Int. J. Pharm. 559, 427–428. Epub 2019/03/02. 10.1016/j.ijpharm.2019.02.037 30819434

[B11] Charoenviriyakul C. Takahashi Y. Nishikawa M. Takakura Y. (2018). Preservation of exosomes at room temperature using lyophilization. Int. J. Pharm. 553 (1-2), 1–7. Epub 2018/10/15. 10.1016/j.ijpharm.2018.10.032 30316791

[B12] Chen Y. S. Lin E. Y. Chiou T. W. Harn H. J. (2020). Exosomes in clinical trial and their production in compliance with good manufacturing practice. Ci Ji Yi Xue Za Zhi 32 (2), 113–120. Epub 2020/04/10. 10.4103/tcmj.tcmj_182_19 32269942 PMC7137364

[B13] Choi H. Choi K. Kim D. H. Oh B. K. Yim H. Jo S. (2022). Strategies for targeted delivery of exosomes to the brain: Advantages and challenges. Pharmaceutics 14 (3), 672. Epub 2022/03/27. 10.3390/pharmaceutics14030672 35336049 PMC8948948

[B14] Choi J-W. Lim S. Kang J. H. Hwang S. H. Hwang K-C. Kim S. W. (2020). Proteome analysis of human natural killer cell derived extracellular vesicles for identification of anticancer effectors. Molecules 25 (21), 5216. 10.3390/molecules25215216 33182448 PMC7664935

[B15] Choi S-J. Cho H. Yea K. Baek M-C. (2022). Immune cell-derived small extracellular vesicles in cancer treatment. Bmb Rep. 55 (1), 48–56. 10.5483/BMBRep.2022.55.1.133 34353429 PMC8810553

[B16] Cianga V. A. Campos Catafal L. Cianga P. Pavel Tanasa M. Cherry M. Collet P. (2021). Natural killer cell subpopulations and inhibitory receptor dynamics in myelodysplastic syndromes and acute myeloid leukemia. Front. Immunol. 12, 665541. Epub 2021/05/15. 10.3389/fimmu.2021.665541 33986753 PMC8112610

[B17] Cochran A. M. Kornbluth J. (2021). Extracellular vesicles from the human natural killer cell line Nk3.3 have broad and potent anti-tumor activity. Front. Cell Dev. Biol. 9, 698639. 10.3389/fcell.2021.698639 34368150 PMC8343581

[B18] Coughlan C. Bruce K. D. Burgy O. Boyd T. D. Michel C. R. Garcia-Perez J. E. (2020). Exosome isolation by ultracentrifugation and precipitation and techniques for downstream analyses. Curr. Protoc. Cell Biol. 88 (1), e110. Epub 2020/07/08. 10.1002/cpcb.110 32633898 PMC8088761

[B19] Deng G. Sun Z. Li S. Peng X. Li W. Zhou L. (2018). Cell-membrane immunotherapy based on natural killer cell membrane coated nanoparticles for the effective inhibition of primary and abscopal tumor growth. ACS Nano 12 (12), 12096–12108. Epub 2018/11/18. 10.1021/acsnano.8b05292 30444351

[B20] Di Pace A. L. Tumino N. Besi F. Alicata C. Conti L. A. Munari E. (2020). Characterization of human nk cell-derived exosomes: Role of Dnam1 receptor in exosome-mediated cytotoxicity against tumor. Cancers (Basel) 12 (3), 661. Epub 2020/03/18. 10.3390/cancers12030661 32178479 PMC7140072

[B21] Dosil S. G. Lopez-Cobo S. Rodriguez-Galan A. Fernandez-Delgado I. Ramirez-Huesca M. Milan-Rois P. (2022). Natural killer (nk) cell-derived extracellular-vesicle shuttled micrornas control T cell responses. Elife, 11, e76319 Epub 2022/07/30. 10.7554/eLife.76319 35904241 PMC9366747

[B22] Enomoto Y. Li P. Jenkins L. M. Anastasakis D. Lyons G. C. Hafner M. (2021). Cytokine-enhanced cytolytic activity of exosomes from nk cells. Cancer Gene Therapy. 29, 734, 10.1038/s41417-021-00352-2 34316033 PMC9209332

[B23] Fais S. (2013). Nk cell-released exosomes: Natural nanobullets against tumors. Oncoimmunology 2 (1), e22337. Epub 2013/03/14. 10.4161/onci.22337 23482694 PMC3583913

[B24] Fang F. Xiao W. Tian Z. (2018). Challenges of nk cell-based immunotherapy in the new era. Front. Med. 12 (4), 440–450. Epub 2018/07/27. 10.1007/s11684-018-0653-9 30047028

[B25] Fang F. Xie S. Chen M. Li Y. Yue J. Ma J. (2022). Advances in nk cell production. Cell Mol. Immunol. 19 (4), 460–481. Epub 2022/01/06. 10.1038/s41423-021-00808-3 34983953 PMC8975878

[B26] Farcas M. Inngjerdingen M. (2020). Natural killer cell-derived extracellular vesicles in cancer therapy. Scand. J. Immunol. 92 (4), e12938. 10.1111/sji.12938 32697853

[B27] Federici C. Shahaj E. Cecchetti S. Camerini S. Casella M. Iessi E. (2020). Natural-killer-derived extracellular vesicles: Immune sensors and interactors. Front. Immunol. 11, 262. 10.3389/fimmu.2020.00262 32231660 PMC7082405

[B28] Fernandez-Messina L. Rodriguez-Galan A. de Yebenes V. G. Gutierrez-Vazquez C. Tenreiro S. Seabra M. C. (2020). Transfer of extracellular vesicle-microrna controls germinal center reaction and antibody production. EMBO Rep. 21 (4), e48925. Epub 2020/02/20. 10.15252/embr.201948925 32073750 PMC7132182

[B29] Fu W. Lei C. Liu S. Cui Y. Wang C. Qian K. (2019). Car exosomes derived from effector car-T cells have potent antitumour effects and low toxicity. Nat. Commun. 10 (1), 4355. Epub 2019/09/27. 10.1038/s41467-019-12321-3 31554797 PMC6761190

[B30] Goldenson B. H. Kaufman D. S. (2021). Into the multiverse of gene edited nk cell-based therapeutic strategies. Cell Stem Cell 28 (12), 2041–2043. Epub 2021/12/04. 10.1016/j.stem.2021.11.004 34861144

[B31] Gong J. H. Maki G. Klingemann H. G. (1994). Characterization of a human cell line (Nk-92) with phenotypical and functional characteristics of activated natural killer cells. Leukemia 8 (4), 652–658. Epub 1994/04/01. 8152260

[B32] Gorgens A. Corso G. Hagey D. W. Jawad Wiklander R. Gustafsson M. O. Felldin U. (2022). Identification of storage conditions stabilizing extracellular vesicles preparations. J. Extracell. Vesicles 11 (6), e12238. Epub 2022/06/19. 10.1002/jev2.12238 35716060 PMC9206228

[B33] Groot Kormelink T. Mol S. de Jong E. C. Wauben M. H. M. (2018). The role of extracellular vesicles when innate meets adaptive. Semin. Immunopathol. 40 (5), 439–452. Epub 2018/04/05. 10.1007/s00281-018-0681-1 29616308 PMC6208666

[B34] Gudbergsson J. M. Jonsson K. Simonsen J. B. Johnsen K. B. (2019). Systematic review of targeted extracellular vesicles for drug delivery - considerations on methodological and biological heterogeneity. J. Control Release 306, 108–120. Epub 2019/06/09. 10.1016/j.jconrel.2019.06.006 31175896

[B35] Guo J. Wu C. Lin X. Zhou J. Zhang J. Zheng W. (2021). Establishment of a simplified dichotomic size-exclusion chromatography for isolating extracellular vesicles toward clinical applications. J. Extracell. Vesicles 10 (11), e12145. Epub 2021/09/14. 10.1002/jev2.12145 34514732 PMC8435528

[B36] Han D. Wang K. Zhang T. Gao G. C. Xu H. (2020). Natural killer cell-derived exosome-entrapped paclitaxel can enhance its anti-tumor effect. Eur. Rev. Med. Pharmacol. Sci. 24 (10), 5703–5713. Epub 2020/06/05. 10.26355/eurrev_202005_21362 32495906

[B37] Heinemann M. L. Vykoukal J. (2017). Sequential filtration: A gentle method for the isolation of functional extracellular vesicles. Methods Mol. Biol. 1660, 33–41. Epub 2017/08/23. 10.1007/978-1-4939-7253-1_4 28828646

[B38] Hong Y. Kim I-S. (2022). The therapeutic potential of immune cell-derived exosomes as an alternative to adoptive cell transfer. Bmb Rep. 55 (1), 39–47. 10.5483/BMBRep.2022.55.1.075

[B39] Jaiswal S. Jamieson C. H. Pang W. W. Park C. Y. Chao M. P. Majeti R. (2009). Cd47 is upregulated on circulating hematopoietic stem cells and leukemia cells to avoid phagocytosis. Cell 138 (2), 271–285. Epub 2009/07/28. 10.1016/j.cell.2009.05.046 19632178 PMC2775564

[B40] Jia R. Cui K. Li Z. Gao Y. Zhang B. Wang Z. (2020). Nk cell-derived exosomes improved lung injury in mouse model of Pseudomonas aeruginosa lung infection. J. Physiol. Sci. 70 (1), 50. Epub 2020/10/25. 10.1186/s12576-020-00776-9 33096976 PMC10717361

[B41] Jiang Y. Jiang H. Wang K. Liu C. Man X. Fu Q. (2021). Hypoxia enhances the production and antitumor effect of exosomes derived from natural killer cells. Ann. Transl. Med. 9 (6), 473. 10.21037/atm-21-347 33850870 PMC8039676

[B42] Jong A. Y. Wu C. H. Li J. Sun J. Fabbri M. Wayne A. S. (2017). Large-scale isolation and cytotoxicity of extracellular vesicles derived from activated human natural killer cells. J. Extracell. Vesicles 6 (1), 1294368. Epub 2017/03/23. 10.1080/20013078.2017.1294368 28326171 PMC5345580

[B43] Kaban K. Hinterleitner C. Zhou Y. Salva E. Kantarci A. G. Salih H. R. (2021). Therapeutic silencing of bcl-2 using nk cell-derived exosomes as a novel therapeutic approach in breast cancer. Cancers (Basel) 13 (10), 2397. Epub 2021/06/03. 10.3390/cancers13102397 34063475 PMC8156181

[B44] Kang Y. T. Niu Z. Hadlock T. Purcell E. Lo T. W. Zeinali M. (2021). On-chip biogenesis of circulating nk cell-derived exosomes in non-small cell lung cancer exhibits antitumoral activity. Adv. Sci. (Weinh) 8 (6), 2003747. Epub 2021/03/23. 10.1002/advs.202003747 33747745 PMC7967048

[B45] Kim H. Y. Min H. K. Song H. W. Yoo A. Lee S. Kim K. P. (2022). Delivery of human natural killer cell-derived exosomes for liver cancer therapy: An *in vivo* study in subcutaneous and orthotopic animal models. Drug Deliv. 29 (1), 2897–2911. Epub 2022/09/08. 10.1080/10717544.2022.2118898 36068970 PMC9467548

[B46] Konoshenko M. Y. Lekchnov E. A. Vlassov A. V. Laktionov P. P. (2018). Isolation of extracellular vesicles: General methodologies and latest trends. Biomed. Res. Int. 2018, 1–27. Epub 2018/04/18. 10.1155/2018/8545347 PMC5831698 29662902

[B47] Korenevskii A. V. Milyutina Y. P. Zhdanova A. A. Pyatygina K. M. Sokolov D. I. Sel'kov S. A. (2018). Mass-spectrometric analysis of proteome of microvesicles produced by nk-92 natural killer cells. Bull. Exp. Biol. Med. 165 (4), 564–571. Epub 2018/08/20. 10.1007/s10517-018-4214-7 30121912

[B48] Kundu S. Gurney M. O'Dwyer M. (2021). Generating natural killer cells for adoptive transfer: Expanding horizons. Cytotherapy 23 (7), 559–566. Epub 2021/01/13. 10.1016/j.jcyt.2020.12.002 33431318

[B49] Kusuma G. D. Barabadi M. Tan J. L. Morton D. A. V. Frith J. E. Lim R. (2018). To protect and to preserve: Novel preservation strategies for extracellular vesicles. Front. Pharmacol. 9, 1199. Epub 2018/11/14. 10.3389/fphar.2018.01199 30420804 PMC6215815

[B50] Langevin S. M. Kuhnell D. Orr-Asman M. A. Biesiada J. Zhang X. Medvedovic M. (2019). Balancing yield, purity and practicality: A modified differential ultracentrifugation protocol for efficient isolation of small extracellular vesicles from human serum. RNA Biol. 16 (1), 5–12. Epub 2019/01/04. 10.1080/15476286.2018.1564465 30604646 PMC6380284

[B51] Laskowski T. J. Biederstadt A. Rezvani K. (2022). Natural killer cells in antitumour adoptive cell immunotherapy. Epub 2022/07/26. 10.1038/s41568-022-00491-0 PMC9309992 35879429

[B52] Lavrik I. N. Krammer P. H. (2012). Regulation of Cd95/fas signaling at the disc. Cell Death Differ. 19 (1), 36–41. Epub 2011/11/15. 10.1038/cdd.2011.155 22075988 PMC3252827

[B53] Lee H. Kang H. Kang M. Han C. Yi J. Kwon Y. (2020). Heterogeneous subcellular origin of exosome-mimetic nanovesicles engineered from cells. ACS Biomater. Sci. Eng. 6 (11), 6063–6068. Epub 2021/01/16. 10.1021/acsbiomaterials.0c01157 33449634

[B54] Leon D. L. Fellay I. Mantel P. Y. Walch M. (2017). Killing bacteria with cytotoxic effector proteins of human killer immune cells: Granzymes, granulysin, and perforin. Methods Mol. Biol. 1535, 275–284. Epub 2016/12/04. 10.1007/978-1-4939-6673-8_18 27914086

[B55] Li D. Wang Y. Jin X. Hu D. Xia C. Xu H. (2020). Nk cell-derived exosomes carry mir-207 and alleviate depression-like symptoms in mice. J. Neuroinflammation 17 (1), 126. 10.1186/s12974-020-01787-4 32321532 PMC7178582

[B56] Li P. Kaslan M. Lee S. H. Yao J. Gao Z. (2017). Progress in exosome isolation techniques. Theranostics 7 (3), 789–804. Epub 2017/03/04. 10.7150/thno.18133 28255367 PMC5327650

[B57] Li Y. Hermanson D. L. Moriarity B. S. Kaufman D. S. (2018). Human ipsc-derived natural killer cells engineered with chimeric antigen receptors enhance anti-tumor activity. Cell Stem Cell 23 (2), 181–192.e5. Epub 2018/08/08. 10.1016/j.stem.2018.06.002 30082067 PMC6084450

[B58] Lian G. Mak T. S. Yu X. Lan H. Y. (2021). Challenges and recent advances in nk cell-targeted immunotherapies in solid tumors. Int. J. Mol. Sci. 23 (1), 164. Epub 2022/01/12. 10.3390/ijms23010164 35008589 PMC8745474

[B59] Lieberman J. (2010). Granzyme a activates another way to die. Immunol. Rev. 235 (1), 93–104. Epub 2010/06/12. 10.1111/j.0105-2896.2010.00902.x 20536557 PMC2905780

[B60] Liu S. Galat V. Galat Y. Lee Y. K. A. Wainwright D. Wu J. (2021). Nk cell-based cancer immunotherapy: From basic biology to clinical development. J. Hematol. Oncol. 14 (1), 7. Epub 2021/01/08. 10.1186/s13045-020-01014-w 33407739 PMC7788999

[B61] Liu W. Li L. Rong Y. Qian D. Chen J. Zhou Z. (2020). Hypoxic mesenchymal stem cell-derived exosomes promote bone fracture healing by the transfer of mir-126. Acta Biomater. 103, 196–212. Epub 2019/12/21. 10.1016/j.actbio.2019.12.020 31857259

[B62] Lugini L. Cecchetti S. Huber V. Luciani F. Macchia G. Spadaro F. (2012). Immune surveillance properties of human nk cell-derived exosomes. J. Immunol. 189 (6), 2833–2842. Epub 2012/08/21. 10.4049/jimmunol.1101988 22904309

[B63] Ma C. Jiang F. Ma Y. Wang J. Li H. Zhang J. (2019). Isolation and detection technologies of extracellular vesicles and application on cancer diagnostic. Dose Response 17 (4), 155932581989100. Epub 2019/12/17. 10.1177/1559325819891004 PMC6902397 31839757

[B64] MacDonald G. Shi L. Vande Velde C. Lieberman J. Greenberg A. H. (1999). Mitochondria-dependent and -independent regulation of granzyme B-induced apoptosis. J. Exp. Med. 189 (1), 131–144. Epub 1999/01/05. 10.1084/jem.189.1.131 9874570 PMC1887691

[B65] Markova K. Mikhailova V. Milyutina Y. Korenevsky A. Sirotskaya A. Rodygina V. (2021). Effects of microvesicles derived from nk cells stimulated with il-1 beta on the phenotype and functional activity of endothelial cells. Int. J. Mol. Sci. 22 (24), 13663. 10.3390/ijms222413663 34948459 PMC8708902

[B66] Mendt M. Kamerkar S. Sugimoto H. McAndrews K. M. Wu C. C. Gagea M. (2018). Generation and testing of clinical-grade exosomes for pancreatic cancer. JCI Insight 3 (8), e99263. Epub 2018/04/20. 10.1172/jci.insight.99263 29669940 PMC5931131

[B67] Neviani P. Wise P. M. Murtadha M. Liu C. W. Wu C. H. Jong A. Y. (2019). Natural killer-derived exosomal mir-186 inhibits neuroblastoma growth and immune escape mechanisms. Cancer Res. 79 (6), 1151–1164. Epub 2018/12/14. 10.1158/0008-5472.CAN-18-0779 30541743 PMC6428417

[B68] Nie W. Fan W. Jiang A. Wu G. Liu H. Huang L-L. (2021). Natural killer cell-derived extracellular vesicle significantly enhanced adoptive T cell therapy against solid tumors via versatilely immunomodulatory coordination. Sci. China-Chemistry 64 (11), 1999–2009. 10.1007/s11426-021-1085-8

[B69] Oh S. Lee J. H. Kwack K. Choi S. W. (2019). Natural killer cell therapy: A new treatment paradigm for solid tumors. Cancers (Basel) 11 (10), 1534. Epub 2019/10/17. 10.3390/cancers11101534 31614472 PMC6826624

[B70] Ou Y. H. Zou S. Goh W. J. Wang J. W. Wacker M. Czarny B. (2021). Cell-derived nanovesicles as exosome-mimetics for drug delivery purposes: Uses and recommendations. Methods Mol. Biol. 2211, 147–170. Epub 2020/12/19. 10.1007/978-1-0716-0943-9_11 33336276

[B71] Parolini I. Federici C. Raggi C. Lugini L. Palleschi S. De Milito A. (2009). Microenvironmental Ph is a key factor for exosome traffic in tumor cells. J. Biol. Chem. 284 (49), 34211–34222. Epub 2009/10/06. 10.1074/jbc.M109.041152 19801663 PMC2797191

[B72] Patel G. K. Khan M. A. Zubair H. Srivastava S. K. Khushman M. Singh S. (2019). Comparative analysis of exosome isolation methods using culture supernatant for optimum yield, purity and downstream applications. Sci. Rep. 9 (1), 5335. Epub 2019/03/31. 10.1038/s41598-019-41800-2 30926864 PMC6441044

[B73] Pitchaimani A. Nguyen T. D. T. Aryal S. (2018). Natural killer cell membrane infused biomimetic liposomes for targeted tumor therapy. Biomaterials 160, 124–137. Epub 2018/02/07. 10.1016/j.biomaterials.2018.01.018 29407341

[B74] Rafei H. Daher M. Rezvani K. (2021). Chimeric antigen receptor (car) natural killer (Nk)-Cell therapy: Leveraging the power of innate immunity. Br. J. Haematol. 193 (2), 216–230. Epub 2020/11/21. 10.1111/bjh.17186 33216984 PMC9942693

[B75] Richter M. Vader P. Fuhrmann G. (2021). Approaches to surface engineering of extracellular vesicles. Adv. Drug Deliv. Rev. 173, 416–426. Epub 2021/04/09. 10.1016/j.addr.2021.03.020 33831479

[B76] Sakamoto N. Ishikawa T. Kokura S. Okayama T. Oka K. Ideno M. (2015). Phase I clinical trial of autologous nk cell therapy using novel expansion method in patients with advanced digestive cancer. J. Transl. Med. 13, 277. Epub 2015/08/26. 10.1186/s12967-015-0632-8 26303618 PMC4548900

[B77] Sayitoglu E. C. Georgoudaki A. M. Chrobok M. Ozkazanc D. Josey B. J. Arif M. (2020). Boosting natural killer cell-mediated targeting of sarcoma through dnam-1 and Nkg2d. Front. Immunol. 11, 40. Epub 2020/02/23. 10.3389/fimmu.2020.00040 32082316 PMC7001093

[B78] Shah N. N. Baird K. Delbrook C. P. Fleisher T. A. Kohler M. E. Rampertaap S. (2015). Acute gvhd in patients receiving il-15/4-1bbl activated nk cells following T-cell-depleted stem cell transplantation. Blood 125 (5), 784–792. Epub 2014/12/03. 10.1182/blood-2014-07-592881 25452614 PMC4311226

[B79] Shen M. Ren X. (2018). New insights into the biological impacts of immune cell-derived exosomes within the tumor environment. Cancer Lett. 431, 115–122. 10.1016/j.canlet.2018.05.040 29857125

[B80] Shoae-Hassani A. Hamidieh A. A. Behfar M. Mohseni R. Mortazavi-Tabatabaei S. A. Asgharzadeh S. (2017). Nk cell-derived exosomes from nk cells previously exposed to neuroblastoma cells augment the antitumor activity of cytokine-activated nk cells. J. Immunother. 40 (7), 265–276. Epub 2017/06/18. 10.1097/CJI.0000000000000179 28622272 PMC7543683

[B81] Sparrow E. Bodman-Smith M. D. (2020). Granulysin: The attractive side of a natural born killer. Immunol. Lett. 217, 126–132. Epub 2019/11/15. 10.1016/j.imlet.2019.11.005 31726187

[B82] Sun H. Shi K. Qi K. Kong H. Zhang J. Dai S. (2019). Natural killer cell-derived exosomal mir-3607-3p inhibits pancreatic cancer progression by targeting il-26. Front. Immunol. 10, 2819. 10.3389/fimmu.2019.02819 31921112 PMC6918866

[B83] Tang X. Yang L. Li Z. Nalin A. P. Dai H. Xu T. (2018). First-in-Man clinical trial of car nk-92 cells: Safety test of Cd33-car nk-92 cells in patients with relapsed and refractory acute myeloid leukemia. Am. J. Cancer Res. 8 (6), 1083–1089. Epub 2018/07/24. 30034945 PMC6048396

[B84] Thakur A. Parra D. C. Motallebnejad P. Brocchi M. Chen H. J. (2022). Exosomes: Small vesicles with big roles in cancer, vaccine development, and therapeutics. Bioact. Mater 10, 281–294. Epub 2021/12/14. 10.1016/j.bioactmat.2021.08.029 34901546 PMC8636666

[B85] Wahlgren J. De L. K. T. Brisslert M. Vaziri Sani F. Telemo E. Sunnerhagen P. (2012). Plasma exosomes can deliver exogenous short interfering rna to monocytes and lymphocytes. Nucleic Acids Res. 40 (17), e130. Epub 2012/05/24. 10.1093/nar/gks463 22618874 PMC3458529

[B86] Wang G. Hu W. Chen H. Shou X. Ye T. Xu Y. (2019). Cocktail strategy based on nk cell-derived exosomes and their biomimetic nanoparticles for dual tumor therapy. Cancers (Basel) 11 (10), 1560. Epub 2019/10/17. 10.3390/cancers11101560 31615145 PMC6827005

[B87] Wang L. Wang Y. Quan J. (2020). Exosomal mir-223 derived from natural killer cells inhibits hepatic stellate cell activation by suppressing autophagy. Mol. Med. 26 (1), 81. Epub 2020/09/03. 10.1186/s10020-020-00207-w 32873229 PMC7465359

[B88] Wang L. Wang Y. Quan J. (2020). Exosomes derived from natural killer cells inhibit hepatic stellate cell activation and liver fibrosis. Hum. Cell 33 (3), 582–589. Epub 2020/05/26. 10.1007/s13577-020-00371-5 32449114

[B89] Weng J. Xiang X. Ding L. Wong A. L. Zeng Q. Sethi G. (2021). Extracellular vesicles, the cornerstone of next-generation cancer diagnosis? Semin. Cancer Biol. 74, 105–120. Epub 2021/05/15. 10.1016/j.semcancer.2021.05.011 33989735

[B90] Whitford W. Guterstam P. (2019). Exosome manufacturing status. Future Med. Chem. 11 (10), 1225–1236. Epub 2019/07/10. 10.4155/fmc-2018-0417 31280675

[B91] Wu C-H. Li J. Li L. Sun J. Fabbri M. Wayne A. S. (2019). Extracellular vesicles derived from natural killer cells use multiple cytotoxic proteins and killing mechanisms to target cancer cells. J. Extracell. Vesicles 8 (1), 1588538. 10.1080/20013078.2019.1588538 30891164 PMC6419691

[B92] Wu Y. Deng W. Klinke D. J. (2015). Exosomes: Improved methods to characterize their morphology, RNA content, and surface protein biomarkers. Analyst 140 (19), 6631–6642. Epub 2015/09/04. 10.1039/c5an00688k 26332016 PMC4986832

[B93] Yang D. Zhang W. Zhang H. Zhang F. Chen L. Ma L. (2020). Progress, opportunity, and perspective on exosome isolation - efforts for efficient exosome-based theranostics. Theranostics 10 (8), 3684–3707. Epub 2020/03/25. 10.7150/thno.41580 32206116 PMC7069071

[B94] Yang P. Cao X. Cai H. Feng P. Chen X. Zhu Y. (2021). The exosomes derived from car-T cell efficiently target mesothelin and reduce triple-negative breast cancer growth. Cell Immunol. 360, 104262. Epub 2020/12/30. 10.1016/j.cellimm.2020.104262 33373818

[B95] Yang P. Peng Y. Feng Y. Xu Z. Feng P. Cao J. (2021). Immune cell-derived extracellular vesicles - new strategies in cancer immunotherapy. Front. Immunol. 12, 771551. 10.3389/fimmu.2021.771551 34956197 PMC8694098

[B96] Yu L. L. Zhu J. Liu J. X. Jiang F. Ni W. K. Qu L. S. (2018). A comparison of traditional and novel methods for the separation of exosomes from human samples. Biomed. Res. Int. 2018, 1–9. Epub 2018/08/28. 10.1155/2018/3634563 PMC6083592 30148165

[B97] Yu S. Kim V. N. (2020). A tale of non-canonical tails: Gene regulation by post-transcriptional rna tailing. Nat. Rev. Mol. Cell Biol. 21 (9), 542–556. Epub 2020/06/03. 10.1038/s41580-020-0246-8 32483315

[B98] Zhang L. Y. Yang X. Wang S. B. Chen H. Pan H. Y. Hu Z. M. (2020). Membrane derived vesicles as biomimetic carriers for targeted drug delivery system. Curr. Top. Med. Chem. 20 (27), 2472–2492. Epub 2020/09/24. 10.2174/1568026620666200922113054 32962615

[B99] Zhang M. Shao W. Yang T. Liu H. Guo S. Zhao D. (2022). Conscription of immune cells by light-activatable silencing nk-derived exosome (lasneo) for synergetic tumor eradication. Adv. Sci. (Weinh) 9, e2201135. Epub 2022/06/07. 10.1002/advs.202201135 35665496 PMC9353410

[B100] Zhang Y. Bi J. Huang J. Tang Y. Du S. Li P. (2020). Exosome: A review of its classification, isolation techniques, storage, diagnostic and targeted therapy Applications&lt. Int. J. Nanomedicine 15, 6917–6934. Epub 2020/10/17. 10.2147/IJN.S264498 33061359 PMC7519827

[B101] Zhang Y. Chopp M. Zhang Z. G. Katakowski M. Xin H. Qu C. (2017). Systemic administration of cell-free exosomes generated by human bone marrow derived mesenchymal stem cells cultured under 2d and 3d conditions improves functional recovery in rats after traumatic brain injury. Neurochem. Int. 111, 69–81. Epub 2016/08/20. 10.1016/j.neuint.2016.08.003 27539657 PMC5311054

[B102] Zhu L. Gangadaran P. Kalimuthu S. Oh J. M. Baek S. H. Jeong S. Y. (2018). Novel alternatives to extracellular vesicle-based immunotherapy - exosome mimetics derived from natural killer cells. Artif. Cells Nanomed Biotechnol. 46 (3), S166–S179. Epub 2018/08/10. 10.1080/21691401.2018.1489824 30092165

[B103] Zhu L. Kalimuthu S. Gangadaran P. Oh J. M. Lee H. W. Baek S. H. (2017). Exosomes derived from natural killer cells exert therapeutic effect in melanoma. Theranostics 7 (10), 2732–2745. Epub 2017/08/19. 10.7150/thno.18752 28819459 PMC5558565

[B104] Zhu L. Kalimuthu S. Oh J. M. Gangadaran P. Baek S. H. Jeong S. Y. (2019). Enhancement of antitumor potency of extracellular vesicles derived from natural killer cells by il-15 priming. Biomaterials 190-191, 38–50. Epub 2018/11/06. 10.1016/j.biomaterials.2018.10.034 30391801

